# Resolving the phylogenetic and taxonomic relationship of Xanthomonas and Stenotrophomonas strains using complete rpoB gene sequence

**DOI:** 10.1371/currents.RRN1239

**Published:** 2011-06-02

**Authors:** Vikas Sharma, Prabhu B. Patil

**Affiliations:** Institute of Microbial Technology (CSIR), Sector 39A, Chandigarh -160036, India

**ABSTRACT**

The phytopathogenic genus *Xanthomonas* comprises numerous species and pathovars described primarily on their host and tissue specificities. *Stenotrophomonas maltophilia*, which is non-phytopathogenic and taxonomically closely related to *Xanthomonas*, has undergone several classifications from *Pseudomonas* to *Xanthomonas* and finally to *Stenotrophomonas*. In this study, we have investigated the phylogenetic and taxonomic status of these members using the complete RNA polymerase beta-subunit (*rpoB*) gene sequences available from their sequenced genomes. Not only did we obtain a phylogenetic tree for xanthomonads, but *rpoB* gene sequence information has also resolved the taxonomic relationship of *X. axonopodis* pathovars, *X. albilineans *and other *Xanthomonas* strains, with the most marked evidence being that *Stenotrophomonas* is  synonymous to *Xanthomonas*. This study has revealed the power and potential of complete *rpoB* gene sequence in taxonomic, phylogenetic and evolutionary studies on *Xanthomonas* and *Stenotrophomonas *generic complex.

**Introduction:**

Resolving the taxonomic and phylogenetic status of plant pathogenic bacteria has been challenging due to the existence of numerous pathovars and species complexes [1]. For example, there are more than 50 pathovars in *Pseudomonas syringae* and more than 140 pathovars in *Xanthomonas* spp. [2] [3] [4] [5]. In particular, the taxonomic status of several strains assigned to species like *X. axonopodis*, *X.* *campestris* and *X. albilineans* remain controversial [6] [7] [8], while there is also a call to assign a separate genus status to *Xanthomonas albilineans* [8].* Stenotrophomonas maltophilia* (Sma) is another interesting member of family *Xanthomonadaceae*, which is not a plant pathogen, but is taxonomically and phylogenetically very closely related to *Xanthomonas* [9]. Originally, *S.maltophilia* was classified as *Pseudomonas maltophilia*, re-classified as *Xanthomonas maltophilia* [10] [11]], and later upgraded to a novel genus and named *Stenotrophomonas maltophilia* [12]. Considering the ecological and economical importance of these xanthomonads, resolving their true relationship or taxonomic status is necessary to understanding their evolution.

Molecular phylogenetics and genome-derived taxonomic criteria are revolutionizing the tree of life in general and bacterial taxonomy in particular [13] [14], the foremost development being the use of 16S rRNA typing to reveal the novelty of a species [15]. However, considering the low rate of evolution of the 16SrRNA gene, and the low resolution at infra-species level, this focus has shifted to using protein-coding genes involved in housekeeping functions [15] [16]. This has led to phylogenomics studies in search of new marker genes for bacterial taxonomy (phylogenetic) studies [17] [18]. One such promising marker is the RNA polymerase beta-subunit (*rpoB*) encoding gene, because its average nucleotide similarity between two bacterial strains was found to correlate with DNA-DNA hybridization (DDH), the traditional gold standard for assigning novel species [18]. Complete *rpoB *gene sequencing has been proposed as a supplement to DDH [18], and for species delineation, *rpoB* gene sequence similarityof ≤ 97.7 % was found to be significantly correlated with DDH value <70 %. Similarly, complete *rpoB* gene sequencesimilarity <85.5 % delineated genera [18]. Complete *rpoB* gene sequencing is emerging as one of the powerful marker genes, owing to its large size (4 kb) and immunity to horizontal gene transfer, as reported in a recent phylogenomic study [17].In the present study we have investigated the phylogenetic and taxonomic relationship among various xanthomonads using the complete *rpoB* gene sequence.

**Results and Discussion:**

Whole-genome sequence of 17 strains of *Xanthomonas* and 3 strains of *Stenotrophomonas* are available in the NCBI genome database [19]. These *Xanthomonas *strains belong to what are classically defined as eight species or nine pathovars, based on host/tissue specificities. Among the three *Stenotrophomonas* strains, two belong to species *S. maltophilia* and one to an unassigned species. The *rpoB* gene is around 4.1 kb, except in *X.fuscans* strains (Table 1). Interestingly, the relatively small of size of 3. 7 kb in Xf strain 10535 and 2.9 kb in Xf strain 11122 is apparently due to frame-shift mutation in this gene. It is present as a single copy gene in all the sequenced *Xanthomonas* genomes. Its G+C content is typical of *Xanthomonas/Stenotrophomonas*, which is 66% (Table 1). A phylogenetic tree based on complete* rpoB *gene sequence-data of *Xanthomonas* and *Stenotrophomonas* strains is shown in Figure 1. The tree is robust with high bootstrap values for all the groupings. As expected the three *Stenotrophomonas* strains make one phylogenetic group, and the other phylogenetic group consists of all *Xanthomonas* strains except *X. albilineans,* which distinctly separates from other groups. In the *Xanthomonas* strains, Xoc and Xoo strains make the first sub-group, the Xcm and Xcv strains make the second sub-group, the third sub-group includes the pathovars Xac, Xp, Xav and Xf, and the Xcc strains make the fourth sub-group. Interestingly, the novel *Xanthomonas* species, Xg and Xv, form independent lineages without any relatives.

**Table1. List of *Xanthomonas* and *Stenotrophomonas* studied and their *rpoB* gene sequence information.**

**Table:** 

**S. No**	**Strain**	**Abbreviation**	**Gene size (bp)**	**G+C %**
1.	*X. oryzae *pv. *oryzae *KACC10331	XooK	4152	61.8%
2.	*X. oryzae pv. oryzae *MAFF 311018	XooM	4152	61.8%
3.	*X. oryzae *pv. *oryzae* PXO99A	XooP	4152	61.7%
4.	*X. oryzae* pv. *oryzicola *BLS256	Xoc	4152	61.8%
5.	*X. campestris* pv. *vasculorum *NCPPB702	Xcv	4152	61.3%
6.	*X. campestris* pv. *musacearum *NCPPB4381	Xcm	4152	61.3%
7.	*X. axonopodis *pv. *citri *str. 306	Xac	4152	62.5%
8.	*Xanthomonas axonopodis *pv. *vesicatoria* str. 85-10	Xav	4152	62.5%
9.	*X.* *fuscans* subsp. *aurantifolii *str. ICPB10535	Xf 10535	3750	63%
10.	*X. fuscans* subsp. *aurantifolii* str. ICPB 11122	Xf 11122	2943	62.8%
11.	*X. campestris pv. campestris *str. B100	XccB	4164	62.3%
12.	*X. campestris pv. campestris* str. ATCC 33913	XccA	4164	62.3%
13.	*X. campestris pv. campestris* str. 8004	Xcc8	4164	62.3%
14.	*X. perforans *str. 91-118	Xp	4152	62.4
15.	*X. gardneri * str. 101	Xg	4152	61.9
16.	*X. vesicatoria *str.1111	Xv	4152	62.6
17.	*Xanthomonas albilineans *	Xalb	4152	61.9%
18.	*Stenotrophomonas maltophilia* K279a	SmaK	4167	64.1%
19.	*Stenotrophomonas maltophilia* R551-3	SmaR	4155	63.9%
20.	*Stenotrophomonas* SKA14	SKA14	4155	64.1%

The* rpoB* gene identity among different *Xanthomonas* and *Stenotrophomonas* strains is shown in Table 2. This study validates the right taxonomic status between *Xanthomonas oryzae* pathovars, (Xoo and Xoc) as they also share 99% sequence identity (well above the cutoff for novel species assignment, which is ≤ 97.7% [18]). However, the species status assigned to the other monocot pathovars, Xcv and Xcm, is under debate, and there has been a call to assign novel species status (*X vasicola*) to these strains [20]. In fact this study also supports assigning novel species status to Xcv and Xcm and removing the species *X. campestris* designation, as the *rpoB* gene sequence identity with the Xcc ATCC33913, the type strain of the species *X campestris* as well as the the pathovar *Xanthomonas campestris* pv. *campestris*, is only 95%. This is well below the cutoff for novel species assignment, which is ≤ 97.7% [18]. Similarly, *rpoB* gene sequence comparison supports classification of two groups of tomato and pepper pathogens in to novel species as *X. vesicatoria* and *X. gardneri* [21] [22]. Both Xv and Xg share only 95-96% sequence identities with rest of *Xanthomonas *strains. This is below the cutoff proposed for novel species assignment, which is ≤ 97.7% [18].

**Figure:**
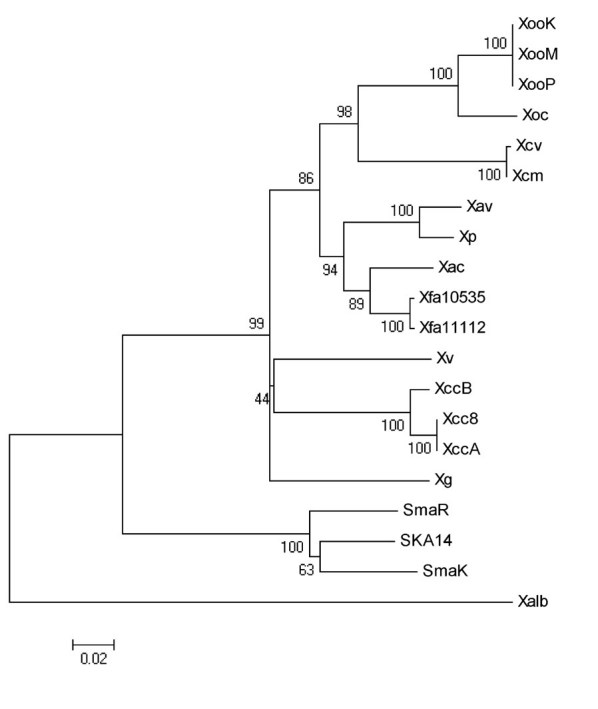
**Figure 1.** Phylogenetic tree of *Xanthomonas *and *Stenotrophomonas *strains based on the complete *rpoB*   gene sequence

One controversial topic has been the relationship between the *X. axonopodis* pathovars, Xac and Xav. Although DNA-DNA hybridization studies suggested that they belong to the same species, and were rightly classified as *X. axonopodis* pathovars [21], other researchers went ahead and classified them into different species, applying more stringent conditions, with Xav becoming *Xanthomonas euvesicatoria* [22]. In this case also, the *rpoB* gene similarity clearly suggests that Xac and Xav are synonymous, as their similarity is 98% (above the cutoff for novel species assignment, which is ≤ 97.7% [18]). Interestingly, some of the *Xanthomonas axonopodis* pv. *vesicatoria* strains (ATCC BAA-983T = NCPPB 4321T) were assigned as novel species, i.e., *Xanthomonas perforans* (Xp) [22].Again, the *rpoB* gene comparison clearly indicates that the classification is incorrect as Xav and Xp share 99% sequence identity (well above the cutoff for novel species assignment, which is ≤ 97.7% [18]).  Similarly, *rpoB* gene sequence comparison shows that *X. fuscans* has been incorrectly assigned a separate species status [23], and that it belongs to the species *X. axonopodis*, as the sequence similarity with other *axonopodis* strains is 98-99% (above the cutoff for novel species assignment, which is ≤ 97.7% [18]).

**Table 2.** Complete *rpoB* gene identity among different *Xanthomonas *and *Stenotrophomonas* strains

**Table:** 

	**XOC**	**XOOK**	**XCM**	**XCV**	**XAC**	**XAV**	**XF**	**XALB**	**SMAK**	**SMAR**	**SKA14**	**XCCA**	**XG**	**XP**	**XV**
**XOC**	4152/4152 (100%)	4099/4152 (99%)	4011/4154 (97%)	4009/4154 (97%)	4011/4153 (97%)	3997/4150 (96%)	3986/4163 (96%)	3683/4185 (88%)	3777/4172 (91%)	3773/4172 (90%)	3778/4172 (91%)	3951/4157 (95%)	3967/4155 (95%)	3998/4150 (96%)	3968/4152 (96%)
**XOOK**	4099/4152 (99%)	4152/4152 (100%)	4010/4153 (97%)	4008/4153 (97%)	4007/4153 (96%)	3999/4150 (96%)	3980/4161 (96%)	3674/4183 (88%)	3777/4173 (91%)	3773/4174 (90%)	3777/4173 (91%)	3950/4156 (95%)	3968/4156 (95%)	4000/4150 (96%)	3967/4153 (96%)
**XCM**	4011/4154 (97%)	4010/4153 (97%)	4152/4152 (100%)	4150/4152 (99%)	4027/4153 (97%)	3999/4152 (96%)	3999/4161 (96%)	3672/4185 (88%)	3777/4170 (91%)	3772/4169 (90%)	3777/4170 (91%)	3948/4156 (95%)	3971/4154 (96%)	4000/4151 (96%)	3976/4152 (96%)
**XCV**	4009/4154 (97%)	4008/4153 (97%)	4150/4152 (99%)	4152/4152 (100%)	4025/4153 (97%)	3997/4152 (96%)	3997/4161 (96%)	3670/4185 (88%)	3775/4170 (91%)	3770/4169 (90%)	3775/4170 (91%)	3946/4156 (95%)	3969/4154 (96%)	3998/4151 (96%)	3974/4152 (96%)
**XAC**	4011/4153 (97%)	4007/4153 (96%)	4027/4153 (97%)	4025/4153 (97%)	4152/4152 (100%)	4069/4151 (98%)	4072/4159 (98%)	3701/4185 (88%)	3794/4172 (91%)	3793/4171 (91%)	3792/4172 (91%)	3962/4157 (95%)	3990/4155 (96%)	4069/4151 (98%)	3994/4152 (96%)
**XAV**	3997/4150 (96%)	3999/4150 (96%)	3999/4152 (96%)	3997/4152 (96%)	4069/4151 (98%)	4152/4152 (100%)	4025/4159 (97%)	3715/4188 (89%)	3794/4174 (91%)	3787/4173 (91%)	3795/4173 (91%)	3961/4160 (95%)	3971/4152 (96%)	4118/4152 (99%)	3986/4154 (96%)
**XF**	3620/3751 (97%)	3617/3751 (96%)	3635/3751 (97%)	3634/3751 (97%)	3704/3750 (99%)	3657/3749 (98%)	3750/3750 (100%)	3333/3778 (88%)	3435/3769 (91%)	3436/3769 (91%)	3430/3769 (91%)	3584/3753 (95%)	3610/3753 (96%)	3661/3749 (98%)	3608/3749 (96%)
**XALB**	3683/4185 (88%)	3674/4183 (88%)	3673/4186 (88%)	3671/4186 (88%)	3702/4186 (88%)	3716/4189 (89%)	3673/4192 (88%)	4152/4152 (100%)	3693/4181 (88%)	3688/4178 (88%)	3687/4181 (88%)	3694/4185 (88%)	3687/4184 (88%)	3706/4188 (88%)	3689/4189 (88%)
**SMAK**	3787/4182 (91%)	3787/4183 (91%)	3786/4179 (91%)	3784/4179 (91%)	3803/4181 (91%)	3803/4183 (91%)	3779/4191 (90%)	3696/4184 (88%)	4167/4167 (100%)	4079/4167 (98%)	4082/4168 (98%)	3806/4183 (91%)	3810/4189 (91%)	3798/4182 (91%)	3811/4184 (91%)
**SMAR**	3773/4172 (90%)	3774/4175 (90%)	3773/4170 (90%)	3771/4170 (90%)	3794/4172 (91%)	3788/4174 (91%)	3773/4183 (90%)	3687/4177 (88%)	4067/4155 (98%)	4155/4155 (100%)	4066/4156 (98%)	3794/4171 (91%)	3798/4176 (91%)	3783/4171 (91%)	3802/4175 (91%)
**SKA14**	3778/4172 (91%)	3777/4173 (91%)	3777/4170 (91%)	3775/4170 (91%)	3792/4172 (91%)	3795/4173 (91%)	3766/4182 (90%)	3685/4179 (88%)	4070/4156 (98%)	4066/4156 (98%)	4155/4155 (100%)	3804/4169 (91%)	3807/4176 (91%)	3789/4171 (91%)	3811/4174 (91%)
**XCCA**	3960/4166 (95%)	3959/4165 (95%)	3957/4165 (95%)	3955/4165 (95%)	3971/4166 (95%)	3970/4169 (95%)	3952/4175 (95%)	3699/4190 (88%)	3805/4182 (91%)	3804/4181 (91%)	3815/4180 (91%)	4164/4164 (100%)	3998/4168 (96%)	3971/4168 (95%)	3990/4165 (96%)
**XG**	3967/4155 (95%)	3968/4156 (95%)	3971/4154 (96%)	3969/4154 (96%)	3990/4155 (96%)	3971/4152 (96%)	3963/4165 (95%)	3687/4184 (88%)	3797/4176 (91%)	3797/4175 (91%)	3806/4175 (91%)	3986/4156 (96%)	4152/4152 (100%)	3972/4152 (96%)	3990/4151 (96%)
**XP**	3998/4150 (96%)	4000/4150 (96%)	4000/4151 (96%)	3998/4151 (96%)	4069/4151 (98%)	4118/4152 (99%)	4029/4159 (97%)	3705/4187 (88%)	3789/4173 (91%)	3783/4171 (91%)	3789/4171 (91%)	3962/4159 (95%)	3972/4152 (96%)	4152/4152 (100%)	3991/4154 (96%)
**XV**	3968/4152 (96%)	3967/4153 (96%)	3976/4152 (96%)	3974/4152 (96%)	3994/4152 (96%)	3986/4154 (96%)	3961/4162 (95%)	3688/4188 (88%)	3800/4173 (91%)	3799/4172 (91%)	3808/4171 (91%)	3981/4156 (96%)	3990/4151 (96%)	3991/4154 (96%)	4152/4152 (100%)

*Xanthomonas albineans* forms a distinct lineage in the phylogenetic tree based on *rpoB* gene sequence (Fig 1), and has been rightly assigned to a novel species taxonomically, which is supported by *rpoB *sequence similarity of only 88% with other *Xanthomonas *strains (well below the cutoff for novel species assignment, which is ≤ 97.7% [18]). Surprisingly, there have also been attempts/suggestions to put it into a novel genus [8]. The *rpoB* similarity values here support the argument that *X. albilineans* is indeed a distinct species but not a novel genus, because the sequence similarity of its *rpoB* gene with that of the sequenced *Xanthomonas* strains is 88% (which is well above the cutoff for novel genus assignment based on *rpoB *gene sequence, which is <85.5% [18]). Similarly, *Stenotrophomonas maltophilia* was originally classified as *Pseudomonas maltophilia* [10], then re-classified as *Xanthomonas maltophilia*, only to be again classified to a novel genus i.e. *Stenotrophomonas maltophilia* [11] [12]. However our study suggest that *Stentrophomonas *was rightly classified as, and is synonymous to *Xanthomonas*, because the sequence similarity of its *rpoB* gene with that of sequenced *Xanthomonas* strains is 91% (which is well above the cutoff for novel genus assignment, which is <85.5% [18]). In fact, the *rpoB* gene of *Stenotrophomonas* strains show more (91%) sequence identity with all the sequenced *Xanthomonas* strains than *X. albilineans*, which shows less (88%) sequence identity with all other sequenced *Xanthomonas* strains.

Overall, our study reveals the power of *rpoB* gene sequencing in resolving the phylogeny and taxonomy of xanthomonads. The first insight being that *X. axonopodis* species members, Xac and Xav are indeed pathovars of same species, as they had been originally classified. The* rpoB *gene sequence comparisons also support classification of Xcm/Xcv as novel species ie *X .vasicola,* and also of *X. albilineans *as its present novel species of *Xanthomonas,* but not as a novel genus. The most surprising revelation is that *Stenotrophomonas *is synonymous to *Xanthomonas*, as previously classified by the Swings group [11]. The highlight of this study is that using the complete *rpoB* gene sequence information is useful for the phylogenetic and taxonomic studies of existing and novel strains of *Xanthomonas*. 

We would like to point out the fact the *rpoB* gene sequence constitutes only a part of the valuable (genomic) data, and that it is not the final say on the phylogenetic and taxonomic status of xanthomonad strains, particularly, when complete genome sequencing is becoming more economical, and with the availability of highly robust genome based criterions for phylogeny and taxonomy like Average Nucleotide Identity [24]and Digital DNA-DNA hybridization [25]. However, *rpoB* gene information is also promising as a simple, supplementary and valuable aid in attempts towards understanding the phylogenetic and taxonomic relationship of a complex group of plant-associated bacteria like *Xanthomonas*.

**Materials and Methods:**

Complete *rpoB* gene sequences were retrieved from the respective genome of *Xanthomonas* and *Stenotrophomonas* strains available at NCBI [19]. Phylogenetic tree was built using MEGA5  [26] using NJ method, with 1000 bootstrap replicates. Sequence comparisons were obtained by NCBI BlastN [27].

**Acknowledgments**

We thank Drs. BD Shenoy, Suresh Korpole, A. Pinnaka for their comments and discussions on the manuscript.

**Funding information**

This work was financially supported by Council for Scientific and Industrial Research (CSIR-IMTECH). VS is currently a research intern under Diamond Jubilee Scheme of CSIR at IMTECH. 

**Competing interests**

The authors have declared that no competing interests exist.
